# Persistent menstruation in transgender people using testosterone gender-affirming hormone therapy

**DOI:** 10.1080/26895269.2024.2403140

**Published:** 2024-09-17

**Authors:** Sav Zwickl, Alex Fang Qi Wong, Laura Burchill, Shalem Y. Leemaqz, Teddy Cook, Lachlan M. Angus, Kalen Eshin, Charlotte V. Elder, Sonia R. Grover, Ada S. Cheung

**Affiliations:** a Trans Health Research Group, Department of Medicine (Austin Health), The University of Melbourne, Melbourne, Victoria, Australia; b College of Medicine and Public Health, Flinders University, Adelaide, South Australia, Australia; c The Kirby Institute, University of New South Wales, Sydney, New South Wales, Australia; d Department of Endocrinology, Austin Health, Melbourne, Victoria, Australia; e Department of Community and Clinical Health, La Trobe University, Melbourne, Victoria, Australia; f Department of Paediatrics, The University of Melbourne, Melbourne, Victoria, Australia; g Department of Paediatric and Adolescent Gynaecology, Royal Children’s Hospital, Melbourne, Victoria, Australia; h Department of Gynaecology, Mercy Hospital for Women, Melbourne, Victoria, Australia

**Keywords:** Androgens, menstruation, testosterone, transgender persons

## Abstract

**Background:**

The cessation of menstruation is a common and desired outcome of testosterone gender-affirming hormone therapy (GAHT), for trans and gender diverse (collectively referred to as trans) individuals recorded female at birth. This study aimed to (a) assess the prevalence and characteristics of menstruation before and after commencing testosterone GAHT, and (b) explore factors associated with persistent menstruation.

**Method:**

Trans people using testosterone therapy for gender affirmation, aged ≥16 years and living in Australia completed an online cross-sectional survey between 28 August to 31 December 2020. Multivariable logistic regression analysis was applied to estimate the effect size of factors potentially contributing to the cessation or persistence of menstruation 6 months after starting testosterone therapy.

**Results:**

There were 401 eligible respondents. Median age for commencement of testosterone GAHT was 23 years (IQR = 20, 28) and the median duration of testosterone therapy was 37 months (IQR = 19, 65). Intramuscular testosterone undecanoate injections were the most used formulation. Persistent menstruation >6 months after commencing testosterone GAHT was reported by 87 (22.8%) participants. The odds of reporting persistent menstruation >6 months after commencing testosterone were (a) higher in people using testosterone gels/creams compared to testosterone injections (OR = 11.3 [1.1, 118.2]), and (b) lower in individuals with a body mass index ≥30 kg/m^2^ versus <30 kg/m^2^ (OR = 0.5 [0.3, 0.99]). No association was found between persistent menstruation >6 months after commencing testosterone and (a) menstrual regularity prior to commencing testosterone GAHT, and (b) age at commencement of treatment.

**Conclusion:**

Persistent menstruation >6 months after commencing testosterone GAHT may be impacted by body mass index and the mode of testosterone administration. This is important information for individuals considering testosterone GAHT as well as for clinicians advising on the benefits and drawbacks of different forms of testosterone administration as well as the likelihood of menstrual cessation.

## Introduction

Many trans and gender diverse people (hereafter referred to as *trans*), who are recorded female at birth, use testosterone gender-affirming hormone therapy (GAHT). This involves testosterone therapy to facilitate the development of specific physical characteristics such as voice lowering, growth of facial and body hair and increase in muscle bulk, in order to align with a person’s gender and as a result, improve associated gender dysphoria and psychological functioning (Cheung et al., 2019). However, the decision to undertake testosterone GAHT is complex and requires that both the trans individual and treating doctor have an in-depth understanding of the possible benefits, risks, and side-effects (Cheung et al., 2019).

For trans people recorded female at birth, one of the most desired effects of testosterone GAHT is menstrual cessation; menstruation being a common cause of gender dysphoria (Schwartz et al., 2022). Testosterone (and sex steroids) impact menstruation *via* a negative feedback loop, whereby high levels suppress pituitary production of luteinizing hormone (LH) and follicle-stimulating hormone (FSH) which in turn suppress ovulation and menstruation. Yet, changes to the lining of the uterus are not necessarily uniform; post-hysterectomy pathology reports from individuals taking testosterone GAHT reveal a mix of proliferative and atrophic endometria (Hawkins et al., 2021). Endometrial pattern was not found to predict amenorrhea (Hawkins et al., 2021).

Menstrual cessation generally occurs within six months of commencing testosterone GAHT (Ahmad & Leinung, 2017; Defreyne et al., 2020). However, persistent menstruation and breakthrough bleeding are relatively common, though rates of bleeding appear to decrease with increased time using testosterone (Defreyne et al., 2020; Grimstad et al., 2021). Little is known about the factors that may contribute to ongoing bleeding following commencement of testosterone GAHT. As a result, clinicians prescribing this therapy have no means to know which patients are most likely to achieve menstruation cessation, nor how to advise them accordingly.

There is speculation that testosterone dose, formulation, and serum levels may impact menstrual cessation in trans people. However, research is sparse and existing studies have reported conflicting results. For example, Nakamura et al. (2013) found that amenorrhea in trans people was dose-dependent in the early months of testosterone GAHT. However, Ahmad and Leinung (2017) who reported individual variation in length of time taken to reach menstrual cessation, noted that this had a relatively small correlation with the testosterone dose. Both these studies involved intramuscular (IM) testosterone and only one reported serum testosterone concentrations. Meanwhile, Defreyne et al. (2020) found that vaginal bleeding and spotting were associated with lower serum testosterone and using gel formulations (vs. IM testosterone), while Pelusi et al. (2014) reported that the type of formulation (IM vs. gel) didn’t impact time taken to achieve amenorrhea. There is a need for more nuanced and replicated data on the effects of testosterone dose, formulation, and serum levels on cessation of menstruation.

Other factors potentially impacting the cessation of menstruation in individuals taking testosterone GAHT remain elusive, with existing knowledge primarily anecdotal and garnered from repeated clinical encounters. These experiences have suggested that people with irregular menstruation prior to testosterone therapy are more likely to have menstrual cessation, such as individuals with polycystic ovarian syndrome (PCOS). There are also some data suggesting that PCOS and obesity (BMI of >30 kg/m^2^) are more common in trans people recorded female at birth, prior to commencement of testosterone (Baba et al., 2007; Balen et al., 1993; Bosinski et al., 1997; Futterweit et al., 1986). In addition to a higher prevalence of PCOS, those who are obese may also be more prone to secondary hypogonadism which may lead to menstrual irregularity or cessation (Eng et al., 2024). Similarly with advancing age approaching menopause, older individuals may also be more likely to experience menstrual irregularity or menstrual cessation (Bae et al., 2018). Whilst the personal experiences of clinicians may provide the impetus to explore factors potentially contributing to persistence or cessation of menstruation following testosterone GAHT, evidence-based data is needed.

This study aimed to explore the factors associated with persistent menstruation in trans people using testosterone GAHT, aged ≥16 years and living in Australia. It was hypothesized that higher odds of persistent menstruation >6 months after commencing testosterone would be observed in individuals (a) reporting regular or more than monthly menstruation prior to commencing testosterone versus infrequent or less than monthly menstruation, (b) using testosterone gel/cream as opposed to testosterone injections, (c) commencing testosterone at older ages and (d) with a body mass index (BMI) <30 kg/m^2^ compared to a BMI of >30 kg/m^2^.

## Materials and methods

### Design

Data used in this study were collected as part of a larger, Australian longitudinal study known as *TRANSform.* Commencing in May 2020, *TRANSform* has researched health and wellbeing outcomes of trans individuals aged 16 years or older and living in Australia, through a series of sub-studies on key issues. *TRANSform* participants were recruited by non-probability snowball sampling, with invitations to participate posted on social media and shared widely by trans organizations and support groups throughout Australia.

As part of *TRANSform*, participants first completed an enrollment survey that collected an email address and demographic information, including about their history of gender-affirming hormone therapy. Participant email addresses were then used to invite them by email to participate in two or three *TRANSform* sub-studies each year. One such sub-study investigated pelvic pain in individuals using testosterone and has been described elsewhere (Zwickl et al., 2023). In brief, *TRANSform* participants who indicated that they were using testosterone therapy for gender affirmation (*n* = 670) were invited to participate in an online survey titled “Pain experiences in trans men and trans masculine people using testosterone survey.” The email invitation encouraged participation from both people who did and did not experience pain. The survey could be accessed from August 28, 2020 to December 31, 2020.

A subset of data from this pelvic pain sub-study was used for purpose of this study; to assess factors impacting menstrual cessation. The current study examined survey data which related to (a) the type and length of testosterone therapy, and (b) experiences of menstruation before and after commencing testosterone GAHT. Consequently, participants in the pelvic pain study were excluded from the analysis if they were using testosterone GAHT for <6 months or not experiencing menstruation prior to commencing testosterone.

### Survey design and data collection

The pelvic pain survey was designed by our research team (SZ, AWFQ, TC, and KE), who are also members of the Australian trans community, in collaboration with clinicians specialized in trans gynaecological health (LB, CVE, and SRG) and endocrinology (LMA and ASC) (Zwickl et al., 2023). In addition to demographic data collected in the *TRANSform* enrollment survey, the pelvic pain survey (see Appendix 1), collected data on participants’ testosterone formulation, dosage, and duration of use; and frequency of menstruation prior to and following commencement of testosterone GAHT. These data were collected and managed using REDCap electronic data capture tools, hosted at The University of Melbourne.

### Ethics

Ethics and governance approval were received from the Austin Health Human Research Ethics Committee (HREC/57155/Austin-2019), ACON Research Ethics Review Committee (2020/03), and the Thorne Harbor Health Community Research Endorsement Panel (THH/CREP 20-006). The survey preamble explained to participants that completing the survey implied consent.

### Data analysis

Participant characteristics are reported as frequency (percentage) for categorical variables, and mean (standard deviation), or median (interquartile range) as appropriate for not normally distributed data. Multivariable logistic regression analysis was used to estimate the effects of possible factors contributing to persistent menstruation after commencing testosterone GAHT. These factors were selected prior to performing the analysis based on expert opinion due to a lack of published research on this topic. Results are reported as odds ratios (OR) with corresponding 95% confidence intervals (CI). This was a complete case analysis with an alpha level of 5% (*p* < 0.05) considered statistically significant. Statistical analyses were performed using R version 4.2.2 (R Foundation for Statistical Computing, Vienna, Austria).

## Results

A total of 670 trans people recorded female at birth and using testosterone GAHT were invited to complete the pelvic pain survey, of which 506 (75.5%) responded. Duplicates and incomplete responses (*n* = 20) were subsequently removed. Participants who had been using testosterone GAHT for <6 months (*n* = 58) or did not experience menstruation before starting testosterone (*n* = 30) were also removed. The remaining 401 responses formed the cohort for analysis.

### Characteristics of participants

Details of the study sample are outlined in Table 1. Participants (*n* = 401) ranged in age from 16 to 62 years, with a median age of 27 years (IQR 23, 34). Five individuals (1.2%) reported having an innate variation of sex characteristics (often known as *intersex*) and sixteen (4%) were Aboriginal and Torres Strait Islander Peoples. Most participants (*n* = 312, 77.8%) were trans men, and 89 (22.2%) individuals were non-binary. The age of participants at the time of commencing testosterone GAHT ranged from 15 to 58 years, with a median of 23 years (IQR = 20, 28). The duration of time taking testosterone GAHT ranged from 6 to 409 months, with a median duration of 37 months (IQR 19, 65). Consistent use of testosterone was reported by 349 participants (87.3%) and 359 (89.5%) reported using a full testosterone dose, while 24 (6.0%) used a half dose, and six (1.5%) used a quarter dose.

**Table 1.** Characteristics of the study sample.

Characteristic	n	%
Age (*n* = 399)		
20 and under	43	10.8
21–30	217	54.4
31–40	97	24.3
41–50	30	7.5
51 and over	12	3.0
Gender (*n* = 401)		
Man/Trans man	312	77.8
Non-binary	89	22.2
Testosterone Formulation (*n* = 398)^a^		
Testosterone undecanoate (Reandron)	308	77.4
Testosterone enanthate (Primoteston depot)	30	7.5
Testosterone esters (Sustanon 250)	8	2.0
Testosterone 1% gel (Testogel)	39	9.8
Testosterone 2% gel (Testavan)	6	1.5
Testosterone 5% cream (Androforte 5)	9	2.3
Other	4	1.0
Consistency of testosterone use (*n* = 400)		
Consistent use since starting	349	87.3
Regularly stop and start use	9	2.3
Other (e.g. change in dosage or formulation)	40	10.0
Unsure/Prefer not to say	2	0.5
Testosterone dosage (*n* = 401)		
Full dose	359	89.5
Half dose	24	6.0
Quarter dose	6	1.5
Other	12	3.0
Duration of testosterone GAHT (months) (*n* = 400) Median (IQR)	37 (18.3, 65)	

^a^ Multiple responses allowed for this question so total responses do not sum to 100%.

### Menstruation before and after commencing testosterone

Prior to commencing testosterone GAHT, participants reported experiencing regular monthly menstruation (*n* = 306, 76.3%), bleeding less than monthly (*n* = 73, 18.2%), or bleeding more often than monthly (*n* = 22, 5.5%). Menstruation prior to testosterone was most often described as ‘medium to heavy’ (*n* = 198, 49.7%), followed by ‘light to medium’ (*n* = 116, 29.1%) and ‘very heavy’ (*n* = 63, 15.8%). A majority (*n* = 270, 68%) reported their menstruation prior to testosterone as lasting between 5 to 7 days.

As shown in Table 2, 235 participants (61.7%) reported menstrual cessation within the first three months of testosterone GAHT, and a further 59 (15.5%) reported cessation between three and six months of treatment. Just over one in five participants (*n* = 87, 22.8%) reported that menstruation had continued beyond the first 6 months of testosterone GAHT. At the time of the survey, 45 participants (11.3%), with a median duration of testosterone therapy of 33 months (IQR 17, 56) reported ongoing menstruation. ‘Regular monthly bleeding’ (*n* = 16, 1.6%) and ‘occasional bleeding’ (*n* = 15, 1.5%) were most common, and four participants (0.4%) reported ‘ongoing bleeding or bleeding more often than not’.

**Table 2.** Characteristics of menstruation after commencing testosterone GAHT (*n* = 381).

Characteristic	*n*	%
Menstrual cessation after commencing testosterone		
1–3 months	235	61.7
3–6 months	59	15.5
6+ months/ongoing	87	22.8
Bleeding patterns in individuals with ongoing menstruation		
Occasional spotting	9	0.9
Regular spotting	1	0.1
Occasional bleeding	15	1.5
Regular bleeding (similar to monthly menstruation)	16	1.6
Bleeding more often than not/ongoing bleeding	4	0.4

*Note:* Respondents who indicated ‘unsure/prefer not to say’ regarding menstrual cessation have been excluded from analysis.

### Associated factors

Multivariable logistic regression demonstrated that there were higher odds of reporting persistent menstruation >6 months after commencing testosterone in people using testosterone gels/creams (OR = 11.3 [1.1, 118.2]) compared to testosterone injections, and lower odds in individuals with a body mass index ≥30 kg/m^2^ (OR = 0.5 [0.3, 0.99]) compared to those <30 kg/m^2^. No association was found between persistent menstruation >6 months after commencing testosterone and either menstrual regularity prior to commencing testosterone GAHT or age at commencement of treatment. For details, see Table 3.

**Table 3.** Factors associated with persistent menstruation 6+ months on testosterone GAHT.

Factors	Menstrual cessation <6 months (*n* = 294) *n* (%)	Persistent menstruation >6 months (*n* = 87) *n* (%)	Odds ratio (95% CI)*	*p*-value*
Menstrual regularity prior to testosterone GAHT				
Monthly	225 (76.5)	68 (78.2)	Reference	
More than monthly	15 (5.1)	5 (5.7)	1.3 (0.5, 4.0)	0.6
Less than monthly	54 (18.4)	14 (16.1)	0.9 (0.5, 1.9)	0.8
Testosterone formulation				
Testosterone Injections**^†^**	266 (90.5)	60 (69.0)	2.0 (0.2, 20.2)	0.6
Testosterone gels/creams**^‡^**	24 (8.2)	27 (31.0)	11.3 (1.1, 118.2)	**0.04**
Age at commencement of testosterone (years) (*n* = 380)				
*Mean (SD)*	*24.9 (7.3)*	*25.0 (7.4)*		
20 and under	98 (33.4)	27 (31.0)	Reference	
21–30	146 (49.8)	42 (48.3)	1.0 (0.6, 1.8)	1.0
31–40	33 (11.3)	14 (16.1)	1.6 (0.7, 3.5)	0.3
41–50	13 (4.4)	4 (4.6)	1.3 (0.3, 4.9)	0.7
51 and over^#^	3 (1.0)	0 (0.0)	–	
Obesity (*n* = 374)				
Body mass index <30 kg/m^2^	203 (70.5)	67 (77.9)	Reference	
Body mass index ≥30 kg/m^2^	85 (29.5)	19 (22.1)	0.5 (0.3, 0.99)	**0.04**

* Odds ratio (95% CI) and *p*-values from logistic regression mutually adjusted for all factors presented in this table; ^†^Testosterone undecanoate (Reandron) and Testosterone enanthate (Primoteston depot) collapsed with Testosterone esters (Sustanon 250); ^‡^Testosterone 1% gel (Testogel) collapsed with Testosterone 2% gel (Testavan) and Testosterone 5% cream (Androforte 5); ^#^sample size was too small to obtain OR(95% CI) estimate.

## Discussion

### Main findings

This large cross-sectional survey of trans people using testosterone GAHT found that persistent menstruation >6 months after commencing hormone therapy was reported by 22.8% of respondents. The odds of reporting this were over 10 times greater in people using testosterone gels/creams versus injections, and 50% lower in individuals with a body mass index of ≥30 kg/m^2^ versus <30 kg/m^2^. Neither menstrual regularity prior to commencing testosterone GAHT nor age at commencement of this treatment were associated with persistent menstruation >6 months after commencing testosterone. Overall, these results suggest that testosterone formulation and to a lesser extent obesity, may impact the cessation of menstruation in trans people using testosterone for gender affirmation. These findings provide important information to individuals considering testosterone GAHT who desire the cessation of menstruation, as well as the clinicians advising them on the benefits and drawbacks of different forms of testosterone administration, the likelihood of menstrual cessation, and factors which may contribute to persistent menstruation. This study adds to the very limited pool of information available to clinician’s making decisions about testosterone dosing, mode of administration, and/or monitoring.

### Differences based on testosterone formulation

A key finding of this study was that individuals using testosterone gels/creams had significantly higher odds (OR = 11.3 [1.1, 118.2]) of experiencing persistent menstruation >6 months after commencing testosterone GAHT, compared to those using testosterone injections. This is in line with research reporting testosterone gel (versus injection) was associated with vaginal bleeding and spotting (Defreyne et al., 2020). Defreyne et al. (2020) also found that lower serum testosterone levels were associated with vaginal bleeding and spotting. These findings suggest that injections may lead to higher serum testosterone levels than transdermal formulations, thus be more likely to stop menstruation.

Other factors may also be at play. As mentioned earlier, some research has shown that early amenorrhea is frequency and dose-dependent (Nakamura et al., 2013) while others have reported individual variability with minimal correlation between the dose and time taken to achieve menstrual cessation (Ahmad & Leinung, 2017). Collectively, these findings suggest that the relationship between menstrual cessation and testosterone formulation, dose, and serum levels is complex, and there may be factors that have yet to be discovered and understood. The differing responses to treatment also shows that there is no one-size-fits-all model for testosterone GAHT treatment, and like all trans health, individualized treatment is required. From a clinical perspective, this information has implications for starting doses of testosterone and/or using a gel or cream versus injection. However, more research is needed and should include testosterone serum levels to identify the inter-relationships between these factors and direct clinical decision-making.

### BMI

Interestingly, this study found that individuals with a BMI ≥30 kg/m^2^ had lower odds (OR = 0.5 [0.3, 0.99]) of reporting persistent menstruation >6 months after commencing testosterone GAHT compared to those with a BMI <30. Previous studies have been mixed, with Defreyne et al. (2020) also reporting those with persistent menstruation after 12 months of testosterone had a lower body fat percentage, however Grimstad et al. (2021) reported that BMI did not affect vaginal bleeding. One explanation for individuals with a high BMI having lower odds of persistent menstruation is underlying secondary hypogonadism from chronic illness (Seif et al., 2015). It is also possible that PCOS and/or irregular periods prior to starting testosterone, increases the likelihood of menstruation ceasing sooner (Seif et al., 2015). The clinical implications of this finding are limited, given addressing obesity is an essential element of health care.

### Age and menstrual regularity

Not all the hypothesized factors influenced menstrual cessation. This study found that the age at commencement of treatment was not associated with persistent menstruation >6 months after commencing testosterone. This is similar to findings reported by Defreyne et al. (2020) and indicates that the age of commencement of testosterone GAHT does not impact on persistence of menstruation after 6 months. If such findings can be replicated, this may provide important information to patients considering testosterone GAHT, particularly individuals who believe they may be “too old” for this treatment to be of value. This finding regarding age was also of interest given menstruation is known to change with age. However, age may be a potential confounder with the real factor being changes in menstrual regularity across the lifecycle. Yet in this study, no association was found between persistent menstruation >6 months after commencing testosterone and menstrual regularity prior to commencing testosterone GAHT. This was unexpected given irregular menstruation is also associated with PCOS, which may be more common in trans people recorded female at birth, prior to commencement of testosterone (Baba et al., 2007; Balen et al., 1993; Bosinski et al., 1997; Futterweit et al., 1986).

### Other causes and treatments of persistent bleeding

Other strategies exist beyond testosterone GAHT to help individuals achieve menstrual suppression (e.g. other hormonal options such as progesterone, or, definitive and childbearing-precluding surgical treatments like endometrial ablation or hysterectomy) (Schwartz et al., 2019). However, when menstrual cessation is not achieved with testosterone therapy after 12 months or when individuals experience abnormal/altered bleeding patterns, it is important to consider other causes of persistent bleeding (Schwartz et al., 2019). This may include not only testing hormone levels but also, investigating for the presence of non-cancerous pathology (e.g. fibroids, adenomyosis, and infection) as well as endometrial hyperplasia and carcinoma (e.g. cervical and endometrial) (Hawkins et al., 2021; Schwartz et al., 2019). This forms a key part of holistic trans health care; Schwartz et al. (2019) reflect on the importance of preventative screening as well as managing the impacts of gender-affirming treatments. This is especially important given that the long-term health impacts of GAHT testosterone remain uncertain. Furthermore, GAHT may mask symptoms; a majority of individuals experiencing amenorrhea having endometrial proliferation at hysterectomy (Hawkins et al., 2021). More information is needed on the long-term impacts of testosterone on the endometrium itself, as distinct from menstrual outcomes.

### Clinical implications and Future research

This study illustrates the importance of tailored trans health care. The results have potential implications for switching mode of administration (e.g. from transdermal to IM in cases of persistent menstruation) and/or direct individuals toward other forms of menstrual suppression (Berrahou et al., 2022). These findings illustrate the gaps in knowledge that practitioners must navigate and the need for more information. In this marginalized and traditionally hard to reach population, research has been extremely limited. Future research on the impacts of testosterone GAHT on menstrual cessation and endometrial health are needed that include testosterone doses and serum levels, and prospective studies, to eliminate recall bias.

### Strengths and limitations

This study has several limitations. Participants were excluded who did not experience menstruation prior to commencing testosterone which may have inadvertently excluded people with PCOS and/or a high BMI. The findings on the impact of BMI may therefore be underestimations. As blood testosterone levels were not measured, it was not possible to determine whether individuals were within the standard male physiological range, nor whether blood levels correlated with menstrual cessation. This information would strengthen any future research.

Given recruitment involved non-probability snowball sampling, the cohort may not represent the greater trans population. As the survey was conducted online, this may bias toward respondents who are younger and have access to technology. Recall bias may have also impacted findings, including the accuracy of recollection of dose of testosterone and time to menstrual suppression, particularly in those participants who had been using testosterone for a significant amount of time. Furthermore, the survey was also cross-sectional, thus causation cannot be inferred.

Despite the limitations, the response rate for this survey was very high (75.5%), which we attribute to this being a trans-led study that was responsive to a community priority issue, and our commitment to rapport-building with the Australian trans community. To date this is the largest study exploring menstrual cessation and patterns in trans people using testosterone therapy and adds to limited existing data on this topic.

### Conclusions

Persistent menstruation >6 months after commencing testosterone GAHT may be impacted by mode of testosterone administration and body mass index. This is important information for individuals considering testosterone GAHT and for clinicians weighing the benefits and drawbacks of different forms of testosterone administration. More evidence is needed to support clinical practice, so clinicians can tailor testosterone dose and formulation to patients’ individual needs, health comorbidities, and desired outcomes.

## Appendix 1

### Online survey – pain experiences in trans men and trans masculine people using testosterone

The following survey will be asking directly about experiences of pain related to genitals, ovaries, menstruation and testosterone. Thank you for participating in this research and please seek support if you become distressed. All questions are optional and you can return to this survey if you need some time. 

### Testosterone use

This first set of questions will ask you about your testosterone use and levels. 

1. What date (approximately) did you start testosterone therapy? [dd/mm/yyyy] 
2. What type of testosterone are you currently using? (please select all that apply) 
  - Injection – Reandron 
  - Injection – Primoteston 
  - Injection – Sustanon 
  - Gel/cream – Testogel 
  - Gel/Cream – Androforte 
  - Implant  
  - Other 
  - Unsure/Prefer not to say 
    You selected ‘other’. Can you please elaborate? [free-text] 
3. What were your testosterone levels on your most recent blood test? [nmol/L] 
4. How have you been using testosterone? 
  - I have used testosterone consistently since I started 
  - I regularly stop and start testosterone 
  - Other 
  - Unsure/Prefer not to say 
    You selected “other.” Can you please elaborate? [free-text] 
5. What dose of testosterone are you on? 
  - Full dose 
  - Half dose 
  - Quarter dose 
  - Other (e.g. change between full dose and lower dose) 
  - Unsure/Prefer not to say 
    You selected “other.” Can you please elaborate? [free-text]  

### Pelvic pain before testosterone

The next set of questions will ask you about your experiences of pelvic pain before starting testosterone, including questions related to menstruation. We understand that some of the terminology or subject matter may make some people uncomfortable. Please skip any questions you do not want to answer. 

1. Did you ever experience pelvic pain prior to starting testosterone therapy?  
  - Yes 
  - No 
  - Unsure/Prefer not to say 
2. Did you have periods/bleeding before starting testosterone? 
  - Yes, monthly 
  - Yes, more often than monthly 
  - Sometimes/Occasionally (less often than monthly) 
  - Never 
  - Unsure/Prefer not to say 
    If yes or sometimes to periods, before starting testosterone, on average how many days did your periods/bleeding go for? 
  - 1–2 days 
  - 3–4 days 
  - 5–6 days 
  - 6–7 days 
  - 8+ days 
    If yes or sometimes to periods, before starting testosterone, how would you describe your average period/bleeding? 
  - Very light 
  - Light to medium 
  - Medium to heavy 
  - Very heavy 
    If yes or sometimes to periods, did you experience pain around the time of bleeding/periods? (i.e., in the couple days before or during bleeding/periods) 
  - Always or almost always 
  - Sometimes 
  - Never 
  - Unsure/Prefer not to say 
    If always or sometimes to pain around periods, on a scale of 0–10 (10 = most severe pain) how severe was this pain when it occurred?  
    If yes or sometimes to periods, did you experience pelvic pain between bleeding/periods?  
  - Always or almost always 
  - Sometimes 
  - Never
  - Unsure/Prefer not to say 
    If always or sometimes, on a scale of 0–10 (10 = most severe pain) how severe was this pain when it occurred?  
    If yes or sometimes to periods, did you experience pelvic pain at or around the time of ovulation?  
  - Always or almost always 
  - Sometimes 
  - Never 
  - Unsure/Prefer not to say 
    If always or sometimes, on a scale of 0–10 (10 = most severe pain) how severe was this pain when it occurred? 
    If yes or sometimes to pain before testosterone, did you use medications to suppress periods or reduce pelvic pain?  
  - Yes 
  - No 
  - Unsure/Prefer not to say 
    If yes to medication, what pain relief or medications did you use?  (select all that apply) 
  - Hormonal 
  - Pain killers 
  - Cannabis 
  - Other 
  - Unsure/Prefer not to say 
    You selected “other.” Can you please elaborate? [free-text] 
    If hormonal, what sort of hormonal therapy? [free-text] 
    If hormonal, did the hormonal therapy work? 
  - Yes 
  - Sometimes 
  - No 
  - Unsure/Prefer not to say 
    If pain killers, what sort of pain killers? [free-text] 
    If pain killers, did the pain killers work? 
  - Yes 
  - Sometimes 
  - No 
  - Unsure/Prefer not to say 
  - If cannabis, did cannabis work?Yes
  - Sometimes 
  - No 
  - Unsure/Prefer not to say 
    If pain killers of cannabis, where did you source this pain relief? (please select all that apply) 
  - Prescription from a doctor 
  - Over the counter 
  - Friends 
  - The internet 
  - A dealer 
  - Other 
  - Unsure/Prefer not to say 
    You selected “other.” Can you please elaborate? [free-text] 

### Pelvic pain since starting testosterone

The next set of questions will ask you about your experiences of pelvic pain since starting testosterone, including questions related to menstruation. We understand that some of the terminology or subject matter may make some people uncomfortable. Please skip any questions you do not want to answer. 

1. Have you experienced pelvic pain since commencing testosterone therapy? 
  - Always or almost always 
  - Sometimes 
  - Never 
  - Unsure/Prefer not to say 
    If always or sometimes pain on testosterone, on a scale of 0 to 10 (10 = most severe pain) how severe is your current pelvic pain?  
    If always or sometimes pain on testosterone, are there any triggers for your pelvic pain? (for example, bleeding, exercise) [free-text] 
    If always or sometimes pain on testosterone, is there a time relationship to last dose of testosterone? [free-text] 
    If always or sometimes pain on testosterone, what treatment or strategies (if any) have eased your pelvic pain? (please select all that apply) 
  - Hormonal medication 
  - Pain killers 
  - Cannabis 
  - Heat 
  - Exercise 
  - Other 
  - Unsure/prefer not to say 
    If always or sometimes pain on testosterone, what treatments or strategies have you tried that have not worked? (please select all that apply) 
  - Hormonal medication 
  - Pain killers 
  - Cannabis 
  - Heat 
  - Exercise 
  - Other 
  - Unsure/prefer not to say 
2. Have your periods/bleeding stopped since starting testosterone? 
  - Yes 
  - No 
  - Unsure/Prefer not to say 
    If yes periods stopped, how long did it take your periods/bleeding to stop after starting testosterone? 
  - 1-3 months 
  - 3-6 months 
  - 6-9 months 
  - 9-12 months 
  - Longer than 12 months  
    If no to periods stopping, how would you best describe the bleeding/periods you have experienced since starting testosterone? 
  - Occasional spotting 
  - Regular spotting 
  - Occasional bleeding 
  - Regular bleeding (similar to a monthly period) 
  - Bleeding more often than not/ongoing bleeding 
    If no to periods stopping, have you been offered any options to stop the bleeding/periods? 
  - Yes 
  - No 
  - Unsure/Prefer not to say 
    If yes, can you describe the option/s you have been offered to stop the bleeding/periods? [freetext] 
3. Do you experience genital dryness? 
  - Always 
  - Sometimes 
  - Never 
  - Unsure/Prefer not to say 
    If pain before and on T, how does the pelvic pain you experience using testosterone compare to the pelvic pain you experienced prior to starting testosterone? (0= much less severe, 10= much more severe)  

### Sexual activity and pelvic pain

This next set of questions asks you about your sexual activity since starting on testosterone. We understand that some of the terminology or subject matter may make some people uncomfortable. Please skip any questions you do not want to answer. 

1. Are you sexually active (including masturbation)?  
  - Yes 
  - No 
  - Unsure/Prefer not to say 
    If yes to sexual activity, does touching of your external genitalia cause pain?  
  - Always or almost always 
  - Sometimes 
  - Never 
  - Unsure/Prefer not to say 
    If always or sometimes to pain with external touch, is this pain the same as the pelvic pain? 
  - Yes 
  - Sometimes 
  - No 
  - Unsure/Prefer not to say 
    If yes to sexual activity, do penetrative sexual activities, provoke pain?  
  - Always or almost always 
  - Sometimes 
  - Never 
  - Unsure/Prefer not to say 
  - Don’t do penetrative sexual activities 
    If always or sometimes to pain with penetration, is this pain the same as the pelvic pain?  
  - Yes 
  - Sometimes 
  - No 
  - Unsure/Prefer not to say 
    If yes to sexual activity, does orgasm cause pain? 
  - Always or almost always 
  - Sometimes 
  - Never 
  - Unsure/Prefer not to say 
    If yes to pain with orgasm, is this pain the same as the pelvic pain?  
  - Yes 
  - Sometimes 
  - No 
  - Unsure/Prefer not to say 
    If yes to sexual activity and pelvic pain on testosterone, does the pelvic pain stop you or make you consider alternative ways/methods of being sexually active? 
  - Always or almost always 
  - Sometimes 
  - Never 
  - Unsure/Prefer not to say 

### Pelvic surgeries and diagnoses

The next set of questions asks you about pelvic surgeries and diagnoses. We understand that some of the terminology or subject matter may make some people uncomfortable. Please skip any questions you do not want to answer. 

1. Have you ever had an intrauterine device (IUD)? 
  - Yes 
  - No 
  - Unsure/Prefer not to say 
    If yes to IUD, did the IUD cause/provoke physical pain? 
  - Always or almost always 
  - Sometimes 
  - Never 
  - Unsure/Prefer not to say 
    If yes to pain with IUD, is this pain the same as the pelvic pain?  
  - Yes 
  - No 
  - Unsure/Prefer not to say 
2. Have you ever been diagnosed with endometriosis? 
  - Yes 
  - No 
  - Unsure/Prefer not to say 
    If yes to endometriosis, how was this diagnosed? 
  - No tests, just my pain history  
  - By ultrasound 
  - By laparoscopy  
  - Unsure/Prefer not to say 
    If yes to endometriosis, what date (approximately) was this diagnosed? [dd/mm/yy] 
3. Have you had an ultrasound of your pelvis to look for the cause of the pelvic pain?  
  - Yes 
  - No 
  - Unsure/Prefer not to say 
    If yes to ultrasound, was it reported as normal?  
  - Yes 
  - No 
  - Unsure/Prefer not to say 
    If not normal, what did the ultrasound show? [free-text] 
4. Have you had a hysterectomy (removal of uterus)? 
  - Yes 
  - No 
  - Unsure/Prefer not to say 
    If yes to hysterectomy, what was the reason for the hysterectomy? (please select all that apply) 
  - Gender dysphoria 
  - Pain 
  - Ongoing bleeding 
  - Other  
  - Unsure/Prefer not to say 
    If yes to hysterectomy, what date (approximately) did you have this done? dd/mm/yy 
    If yes to hysterectomy, did hysterectomy improve your pelvic pain?  
  - Yes 
  - No 
  - Unsure/Prefer not to say 
5. Have you had an oophorectomy (removal of ovaries)? 
  - Yes 
  - No 
  - Unsure/Prefer not to say 
    If yes to oophorectomy, what date (approximately) did you have this done? [dd/mm/yy] 
    If yes oophorectomy, did oophorectomy improve your pelvic pain?  
  - Yes 
  - No 
  - Unsure/Prefer not to say 
6. Have you ever been diagnosed with any of the following? (please select all that apply)
  - Vulvodynia 
  - Vaginismus 
  - Polycystic Ovary Syndrome 
  - Genital or Pelvic Cancer 
    If cancer, what type/s of cancer did/do you have? [free-text] 
7. Do you experience regular/recurrent occurrences of any of the following? (please select all that apply) 
  - Vaginal thrush 
  - Bacterial vaginosis 
  - Urinary Tract Infection (UTI) 
8. How many pregnancies have you ever had (including miscarriages and terminations)? 
  - None 
  - One or more 
  - Unsure/Prefer not to say 
    If one or more pregnancies, how many live births have you had? 
  - None 
  - 1 
  - 2 
  - 3 
  - 4 
  - 5 or more 

### Other pain and mental health

The next set of questions will ask you about other pain problems and mental health diagnoses. We understand that some of the terminology or subject matter may make some people uncomfortable. Please skip any questions you do not want to answer. 

1. Do you have any other pain problems?  
  - Yes 
  - No 
  - Unsure/Prefer not to say 
    If yes, please select all that apply. 
  - Migraines 
  - Back pain 
  - Chronic Fatigue 
  - Joint Pain 
  - Fibromyalgia 
  - Other 
2. Have you previously had or currently have a diagnosis of post-traumatic stress disorder?   
  - Current 
  - Previous 
  - Never 
  - Unsure/Prefer not to say 
3. Have you previously had or currently have a diagnosis of depression? 
  - Current 
  - Previous 
  - Never 
  - Unsure/Prefer not to say 
4. Have you previously had or currently have a diagnosis of anxiety?  
  -  Current 
  - Previous 
  - Never 
  - Unsure/Prefer not to say 
5. What is your approximate weight in kg? [30 – 200kg] 
6. What is your approximate height in cm? [100 – 250cm] 
7. Do you have any other comments about your pelvic pain? [free text] 
